# Steroid injections added to the usual treatment of lumbar radicular syndrome: a pragmatic randomized controlled trial in general practice

**DOI:** 10.1186/1471-2474-15-341

**Published:** 2014-10-11

**Authors:** Antje Spijker-Huiges, Jan C Winters, Marten van Wijhe, Klaas Groenier

**Affiliations:** Department of General Practice, University Medical Centre Groningen, Postbus 196, FA20, 9700 AD Groningen, The Netherlands

**Keywords:** Lumbosacral radicular syndrome, Sciatica, General practice, Pain treatment, Epidural injections

## Abstract

**Background:**

Lumbosacral radicular syndrome (LRS) is a self-limiting, benign, painful and impairing condition caused by lumbar disc herniation and inflammatory processes around the nerve root. Segmental epidural steroid injections (SESIs) are helpful to reduce radicular pain on a short-term basis. It is unknown whether SESIs are an effective addition to usual pain treatment of LRS in general practice. In our study, we assessed the effectiveness of SESIs on pain and disability as an addition to usual care for acute LRS in general practice.

**Methods:**

A pragmatic, single-blinded, randomized controlled trial in Dutch general practice was conducted. Circumstances of daily practice were closely followed. Care as usual (CAU) was compared to care as usual combined with an additional SESI in 63 patients in the acute phase of LRS. To detect a minimal clinically important difference of 1.2 points on a numerical rating scale for back pain and a common within-group standard deviation of 1.7 with a two-tailed alpha of 0.05 and a power of 0.80, we needed 33 subjects in each group. Statistical analysis was carried out using mixed models.

**Results:**

A small significant effect in favour of the intervention, corrected for age, sex and baseline values, was found for back pain, impairment and Roland-Morris disability score. The differences, though statistically significant, were too small to be considered clinically relevant. Patients from the intervention group were significantly more satisfied with the received treatment than patients from the control group.

**Conclusion:**

We found a small, statistically significant, but not clinically relevant positive effect of SESIs on back pain, impairment and disability in acute LRS. We do not recommend implementing SESIs as an additional regular treatment option in general practice.

**Electronic supplementary material:**

The online version of this article (doi:10.1186/1471-2474-15-341) contains supplementary material, which is available to authorized users.

## Background

Lumbosacral radicular syndrome (LRS) is defined as pain, radiating from the back into the leg, (“sciatica”) in combination with Lasègue’s sign and/or neurological symptoms originating from a single nerve root. In the Netherlands, LRS is treated by general practitioners (GPs) who adhere to the Dutch College of General Practitioner’s Guideline on LRS. According to this guideline, treatment of LRS consists of pain treatment by taking analgesics as needed, and maintaining normal daily activities as much as possible. The prognosis of LRS is favourable: within eight weeks, 80% of patients have reached bearable pain levels and resumed their work [1]. Some patients, however, do not adequately respond to conservative therapy during and after this period [2]. In 25% of patients, radicular pain becomes chronic [1]. Since there are few effective and evidence-based conservative pain treatments for LRS, caring for these patients can be difficult for GPs [2, 3].

LRS is most commonly caused by protrusion of a lumbar intervertebral disc, which results in an inflammatory response around the nerve root [1, 4]. This inflammatory process is the cause of the radicular pain, rather than mechanical compression [5–8]. Local anti-inflammatory drugs may lessen inflammation and pain, making it easier for patients to profit from the favourable prognosis. Segmental epidural steroid injections (SESIs) and selective nerve root blocks (SNRBs) are examples of local anti-inflammatory treatment. SESIs are not recommended in the Dutch guidelines for general practitioners, but still the intervention is applied as a pain treatment for LRS in the Netherlands.

Efficacy of SESIs in LRS is controversial. Some studies are underpowered, others lack methodological quality to justify definite conclusions [2, 9–12]. In trials that included patients in the acute phase of well-defined radicular syndrome (“sciatica”), SESIs turned out to be more effective than placebo in reducing pain and hastening return to normal daily activities [2, 5, 8, 13–22].

Since patients in the acute phase of LRS are cared for by GPs, SESIs are a possibly useful treatment option in general practice. Most RCTs, however, have been conducted to assess efficacy rather than effectiveness (i.e. placebo-controlled double blinded trials rather than pragmatic trials), in specialist practice, in heterogeneous patient groups, with a short-term follow-up and using a single measuring moment. To our knowledge, no study has assessed effectiveness in general practice, with multiple measuring points and a long term follow-up, in a homogeneous patient group. We assessed the effectiveness of adding SESIs to usual pain treatment for patients with acute LRS in general practice, by means of a pragmatic randomized controlled trial measuring pain, disability and recovery in acute LRS patients with profound sciatica.

## Methods

Our trial took place in and around the city of Groningen, the Netherlands, in 41 general practices with 76 participating GPs. Patients were recruited between January 1^st^ 2005 and December 31^st^ 2007 and followed for one year. This study was approved by the Medical-Ethical Committee of the University Medical Center Groningen in 2005, code 2005/154, and was registered in the Dutch trial register as SLURP, code NTR342. This study was funded by the UMCG, there was no additional funding from external sources.

Our research question calls for a pragmatic study design, which demands that real life conditions are followed as closely as possible. Usual care was therefore not standardized but defined as the treatment decided on by the patients and their GPs. Since Dutch GPs generally adhere to the Dutch College of General Practitioner’s guidelines, usual care consisted of advice and analgesic medication and/or referral as needed [1, 23]. The GPs’ diagnosis of LRS was not evaluated by further specialist physical examination, except to determine the level at which the SESI was to be administered. Patients and caregivers were not blinded.

Inclusion criteria were a diagnosis of LRS established by the GP, complaints of LRS for at least two weeks and no more than four weeks duration and patient age between 18 and 60 years. The upper age limit of 60 was chosen because complications of epidural injections are more common in the over 60 age group, due to osteoporosis. Exclusion criteria were a history of spinal surgery or spinal trauma, maintenance therapy with corticosteroids or anticoagulants, bleeding disorder, cauda equina syndrome, a body mass index of more than 35, inadequate mastery of the Dutch language, allergy to corticosteroids, pregnancy or an active wish to become pregnant, breastfeeding and mental disability. Patients with insulin-dependent diabetes mellitus were not excluded but instructed to measure their serum glucose levels regularly in the 48 hours after the intervention.

Patients in whom the GP established the diagnosis of LRS were given written information on the study, a baseline symptom questionnaire and an informed consent form. Patients were asked to complete the questionnaire and the informed consent form and send them to the research centre. Upon receiving this information, subjects were contacted by the primary researcher to check inclusion and exclusion criteria with a protocolled inclusion form. In the absence of exclusion criteria, the inclusion form was completed.

Randomization was performed by an independent colleague with no further involvement in the study. Pre-prepared, sequentially numbered, opaque, sealed envelopes containing stickers labelled either “SESI” or “CAU”, balanced after 40 assignments, were used. Upon randomisation, the consecutive envelope was opened and the sticker with the allocated treatment was fixed on the completed inclusion form. Inclusion forms, containing personal patient information, were coded and kept separately from follow-up questionnaires. To keep the primary researcher blinded until after the final analysis of the results, follow-up questionnaires were provided with the same codes but contained no personal patient information.

Patients allocated to the intervention group were presented to the department of anaesthesiology of the University Medical Centre Groningen (UMCG). SESIs were administered by a non-involved anaesthesiologist within 48 hours after randomisation. SESIs consisted of 80 milligrams of triamcinolone in 10 millilitres of normal saline and were administered using a lumbar translaminar approach without additional imaging, one level above the presumed LRS in either sitting or lateral position. The skin was anaesthetised with lidocaine, but no local anaesthetics were injected epidurally to avoid problems with mobility and bladder emptying. After the injection, patients were referred back to their GPs for further usual care. When a patient was randomized to the CAU-group the GP provided usual care from the start. The translaminar injection technique without additional imaging, rather than a transforaminal approach with fluoroscopic guidance and administering of local anaesthetics, was chosen because of the pragmatic study design - given the shorter waiting time and better accessibility, the intervention would be applied this way in normal practice as well.

Follow-up in both groups was performed using postal questionnaires regarding pain, disability, and satisfaction with treatment, measured at 2, 4, 6, 13, 26 and 52 weeks after the start of the treatment. The 24-point Roland-Morris Disability Questionnaire (RMDQ) was used for measuring disability [24, 25]. For measuring pain and self-perceived impairment, a numeric rating scale (NRS) from 0 to 10 was used, where 0 meant no pain/impairment and 10 meant the worst pain/impairment imaginable. For measuring satisfaction with treatment, we asked patients to grade their treatment on a scale from 0 to 10, where 0 meant very poor and 10 meant excellent. All variables were measured at every time point. As minimal clinically important differences in the interpretation of the results, a reduction of 30% from baseline was used for the RMDQ-score and 2.0 was used for the NRS pain and impairment scores [24–28].

Power calculations were based on the NRS back pain score at four weeks from the start of the treatment. A difference in NRS back pain score of 1,2 - 2,0 is considered clinically relevant in primary care attendants with low back pain [29–32]. To minimize the risk of underpowering our study, an MCID of 1.2 was used for the sample size calculations. The mean standard deviation of VAS scores in patients with moderate pain is approximately 1,7 [33]. To detect a difference of 1.2 and a common within-group standard deviation of 1.7 with a two-tailed alpha of 0.05 and a power of 0.80, we needed 33 subjects in each group. To compensate for anticipated lost to follow-up, we intended to include 40 subjects in each group.

This study was performed to achieve two goals. The first goal of the study was to test the difference over time between the means of the two treatments. The second goal of the study was to estimate the differences between the means of the two treatments at every time point of measurement. On average, a study of this design would enable us to report the mean difference with a precision (95.0% confidence level) of plus/minus 0.84 points.

All analyses were performed using an intention-to-treat basis. Mixed model regression analysis was performed using SAS 9.2 PROC MIXED. No data imputation is necessary using this model [34]. Patients were a random factor in the model and treatment a fixed factor. For every outcome variable, treatment and time of measurement as independent variables were tested with sex, age and baseline-values as covariates to account for non-balance in the randomization.

## Results

**Figure 1:**
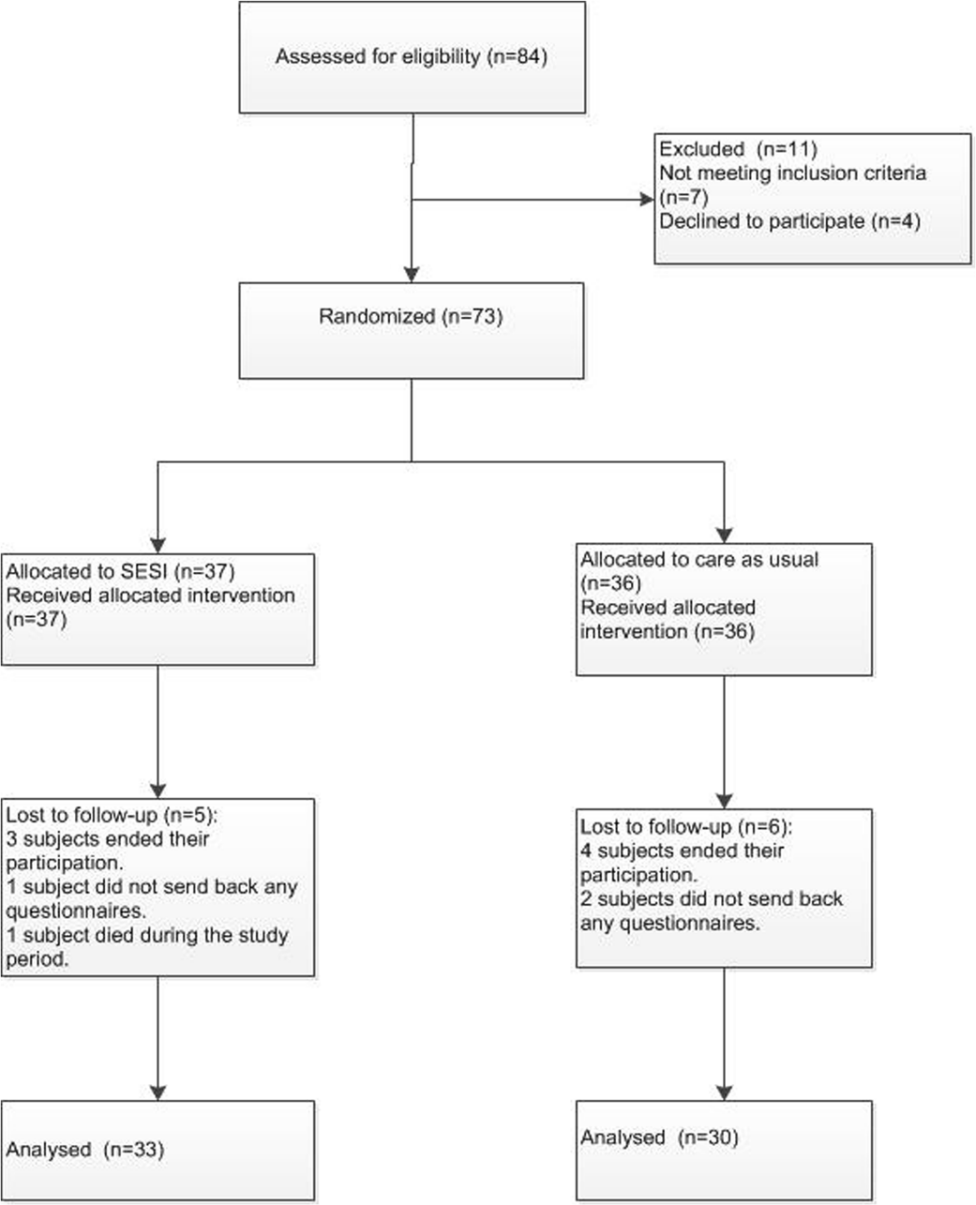
**CONSORT population flow schedule.**

Eighty-four patients were presented to us by their GPs, of whom 73 patients were eligible and included in the study. A flow schedule is presented in Figure 1. Ten randomized patients were not included in the analysis. Of these, seven subjects ended their participation shortly after enrollment and three subjects did not send back any questionnaires despite repeated requests. Of one subject the follow-up was incomplete. She died during the study period due to Burkitt lymphoma, which initially caused radicular pain. The subjects lost to follow-up did not differ significantly in sex, age, randomization group or baseline values from the 63 subjects who were included in the analysis. Of these 63 subjects, 30 were men. The mean age of the study participants at the time of the inclusion was 43,7 years (SD 9,8). The intervention group did not differ significantly from the control group in age or distribution of the sexes.

**Figure 2:**
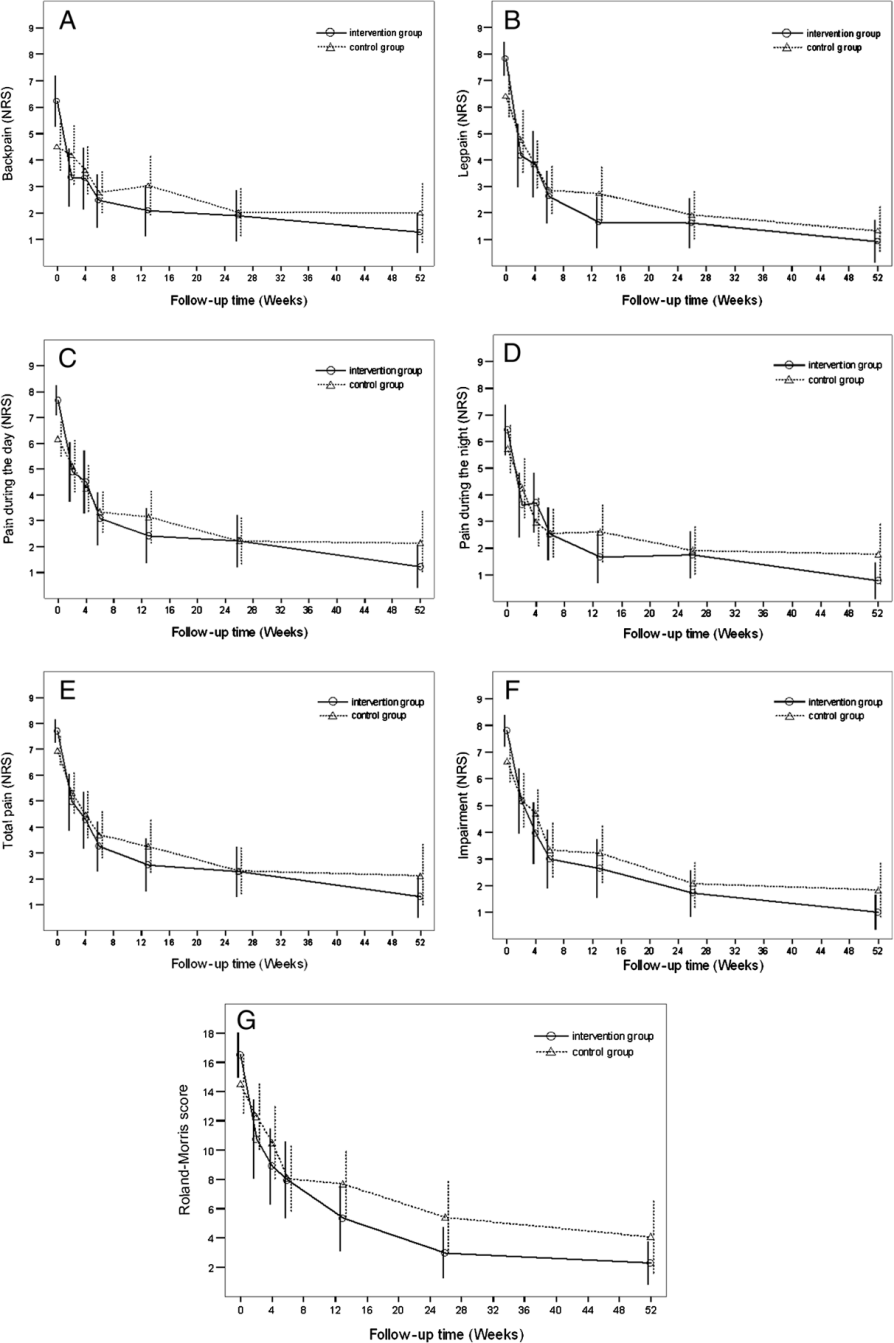
**A**–**G: results of the mixed model analysis for pain, impairment and Roland-Morris disability scores.** When corrected for baseline values, there is a significant effect of the intervention on the RMDQ score and on the NRS scores for back pain and self-perceived impairment (the curve for the intervention group ‘lies below’ the curve for the control group). The effects are statistically significant but too small to be considered clinically relevant. Differences between groups remain constant over the entire study period (the two curves are parallel).

For the 63 study participants, we sent out 441 questionnaires, of which 408 (92,5%) were returned. Means and standard deviations of all variables in both groups for every time point of measurement are presented in Table 1. The intervention group differed significantly from the control group in all baseline values except for leg pain. In the mixed models regression analysis, these differences were corrected for by including the baseline values as a covariate. In Table 2 and in Figure 2A-G, the results of the mixed models regression analysis are shown. Both groups experience a significant decline over time for all symptoms. The intervention group experienced significantly less symptoms than the control group for the RMDQ-score (p = 0,0173), the NRS back pain score (p = 0,0115) and the NRS score for self-perceived impairment (p = 0,0361). These differences between the groups remained constant during the whole follow-up period. In Figure 2A-B, the courses over time of all variables for the entire study period are shown graphically.

**Table 1:** **Mean RMDQ and NRS scores of study participants for every measuring moment in the follow-up period**

Follow-up time (weeks)		0	2	4	6	13	26	52
**RMDQ score**								
	Intervention group mean (SD)	16.5 (4.2)	10.7 (7.1)	8.9 (6.8)	8.0 (6.8)	5.3 (5.9)	3.0 (4.5)	2.3 (3.7)
	Control group mean (SD)	14.5 (6.1)	12.3 (6.1)	10.5 (7.0)	8.1 (6.3)	7.6 (6.3)	5.4 (6.5)	4.1 (6.2)
**NRS back pain**								
	Intervention group mean (SD)	6.2 (2.6)	3.3 (2.9)	3.3 (3.0)	2.5 (2.6)	2.1 (2.5)	1.9 (2.5)	1.3 (1.9)
	Control group mean (SD)	4.5 (2.7)	4.1 (3.0)	3.6 (2.7)	2.8 (2.3)	3.0 (3.0)	2.0 (2.4)	2.0 (2.9)
**NRS leg pain**								
	Intervention group mean (SD)	7.8 (1.7)	4.2 (3.1)	3.8 (3.3)	2.6 (2.5)	1.6 (2.5)	1.6 (2.4)	1.0 (2.0)
	Control group mean (SD)	6.4 (2.3)	4.7 (3.1)	3.9 (2.8)	2.9 (2.5)	2.7 (2.8)	1.9 (2.5)	1.4 (2.2)
**NRS pain during day**								
	Intervention group mean (SD)	7.7 (1.6)	4.9 (3.1)	4.5 (3.2)	3.1 (2.7)	2.4 (2.7)	2.2 (2.6)	1.2 (2.0)
	Control group mean (SD)	6.2 (2.1)	5.1 (2.8)	4.2 (2.6)	3.3 (2.4)	3.1 (2.9)	2.2 (2.3)	2.2 (3.0)
**NRS pain during night**								
	Intervention group mean (SD)	6.4 (2.6)	3.6 (3.2)	3.7 (3.0)	2.5 (2.5)	1.7 (2.6)	1.8 (2.3)	0.8 (1.7)
	Control group mean (SD)	5.7 (2.7)	4.3 (3.0)	3.0 (2.8)	2.6 (2.5)	2.6 (2.9)	1.9 (2.5)	1.8 (2.9)
**NRS total pain**								
	Intervention group mean (SD)	7.7 (1.2)	5.0 (2.9)	4.2 (3.0)	3.3 (2.5)	2.5 (2.5)	2.3 (2.5)	1.3 (2.0)
	Control group mean (SD)	6.9 (1.7)	5.3 (2.6)	4.5 (2.8)	3.7 (2.5)	3.2 (2.8)	2.3 (2.4)	2.1 (3.0)
**NRS impairment**								
	Intervention group mean (SD)	7.8 (1.6)	5.2 (3.2)	4.0 (3.1)	3.0 (2.8)	2.6 (2.9)	1.7 (2.2)	1.0 (1.6)
	Control group mean (SD)	6.7 (2.2)	5.2 (2.8)	4.7 (2.8)	3.3 (2.9)	3.2 (2.9)	2.1 (2.3)	1.9 (2.6)

**Table 2:** **Estimated differences between group means**

Variable	Estimated difference	Standard error	P |t|	95% CI -	95% CI +
**RMDQ-score**	2,5004	1,0435	0,0173	0,4551	4,5456
**NRS back pain**	1,1165	0,4389	0,0115	0,2562	1,9767
**NRS leg pain**	0,6717	0,5100	0,1890	-0,3279	1,6713
**NRS pain during the day**	0,6563	0,5186	0,2068	-0,3601	1,6727
**NRS pain during the night**	0,5285	0,4741	0,2659	-0,4007	1,4577
**NRS total pain**	0,6890	0,4729	0,1463	-0,2378	1,6158
**NRS impairment**	1,0254	0,4867	0,0361	0,0714	1,9793

The differences between group means calculated by the mixed models analysis, over the entire course of the study period using linear regression. In this repeated measures regression analysis, differences between groups are calculated based on the study outcomes, corrected for baseline values, to estimate true values in the random population. We found significant differences between group means for RMDQ-score, NRS back pain score and NRS score for self-perceived impairment. These differences are statistically significant but too small to be considered clinically relevant.

Finally, we found a significant difference in mean patient satisfaction between the two groups. The intervention group rated their treatment 9,0 on a 0 to 10 scale, and the control group rated their treatment 7,2 on a 0 to 10 scale (p = 0,006). No complications or adverse effects of the intervention were reported.

## Discussion

In this study, SESIs yielded a significant overall effect on RMDQ score, back pain, and self-perceived impairment as an additional treatment for LRS in a pragmatic general practice setting. Small differences between pain severity scores and other outcomes, however, may be statistically significant but clinically trivial. As differences of 1.2 – 2.0 on the NRS and a 30% reduction from baseline (which amounts to 4.5 points in our study) in RMDQ score can be considered clinically important to patients, the effects of our intervention are too small to be relevant [24–32].

The intervention group was significantly more satisfied with their treatment than the control group, rating a mean of 9.0 versus 7.2 on a 0 to 10 scale (p = 0,006). As no clinically relevant effect was yielded in our study, the more positive evaluation of the intervention by patients should most probably be attributed to the effect of receiving extra attention and care.

Our study is the first pragmatic trial undertaken in general practice, where most patients with LRS are seen and treated in an early stage. It is one of the few studies aimed to assess effectiveness rather than efficacy of SESIs. To our knowledge this has been done only once before, 15 years ago and in a hospital setting [22]. Outcomes of this study suggested that adding SESIs as a first-line treatment to rest and a nonsteroidal antiinflammatory drug for LRS resulted in additional costs and no gain in efficacy. Our study is the first to evaluate the effect of SESIs on LRS with mixed models multiple regression analysis, which enabled us to assess the effect of this intervention over the whole course of the follow-up time rather than evaluating its effect on a single moment. Whereas most trials in this field are underpowered, we included enough patients to yield a statistically significant effect, although it is still a small patient population.

This study has some possible limitations. One is the fact that the intervention group unfortunately differed significantly from the control group in baseline values. Since randomization was adequately performed, we have no explanation for these differences. In the mixed model regression analysis the baseline differences were corrected for by including the baseline values as a covariate. Baseline differences between groups do, however, raise questions about whether those groups are truly comparable. The MCIDs of measuring instruments may vary between categories of baseline severity in symptoms. According to the literature, comparing our groups was allowed [28, 33]. We are therefore convinced that the difference in baseline values between our study groups are not a problem for the analyses and the ultimate interpretation of our trial results.

It can be argued that for our study goals, the RMDQ-score would have been a more appropriate primary outcome measure than the NRS back pain score. We chose back pain for calculating our sample sizes because the MCID of the NRS back pain score is extensively used and well described in primary care back pain patients [24, 29, 30, 32, 33]. The RMDQ, however, is more responsive in sciatica and might in retrospect have been a better choice as primary outcome measure [24, 25, 28]. Considering the outcomes of the RMDQ scores, we can state that our trial would not have been underpowered had we chosen the RMDQ for calculating the sample sizes.

No adverse effects of our intervention were reported by our subjects. One of the subjects, however, died during our study period due to Burkitt lymphoma, which initially caused radicular pain. Epidural steroids are known to relief symptoms of spinal cord compression caused by tumors or metastases [35]. It is conceivable that administering epidural steroids to a patient whose radicular complaints are caused by cancer, delays diagnosis and treatment. To our knowledge, no reports about this problem have been published.

## Conclusions

Placebo-controlled double blinded randomized trials have yielded positive results for the efficacy as a pain treatment of SESIs on LRS. Our study shows that the intervention has a significant beneficial effect as an additional treatment in general practice as well. This effect however, is too small to be considered clinically relevant to patients. Although our patient sample was small, we do not recommend that administering SESIs for the pain treatment of LRS be implemented as a regular intervention in general practice. Further research should be aimed at adequately treating pain in patients with acute LRS with other interventions.

## Authors’ original submitted files for images

Below are the links to the authors’ original submitted files for images. Authors’ original file for figure 1 Authors’ original file for figure 2 Authors’ original file for figure 3 Authors’ original file for figure 4 Authors’ original file for figure 5 Authors’ original file for figure 6 Authors’ original file for figure 7 Authors’ original file for figure 8

## Abbreviations

SESI: Segmental epidural steroid injection
CAU: Care as usual
LRS: Lumbosacral radicular syndrome
GP: General practitioner
NRS: Numerical rating scale
BMI: Body mass index
MCID: Minimal clinically important difference
SAS: Statistical analysis software
SD: Standard deviation.
