# Supplementary figures and images for: Steroid injections added to the usual treatment of lumbar radicular syndrome: a pragmatic randomized controlled trial in general practice
Source: BMC Musculoskelet Disord. 2014 Oct 11;15:341. doi: 10.1186/1471-2474-15-341 (PMC4200234; doi:10.1186/1471-2474-15-341)

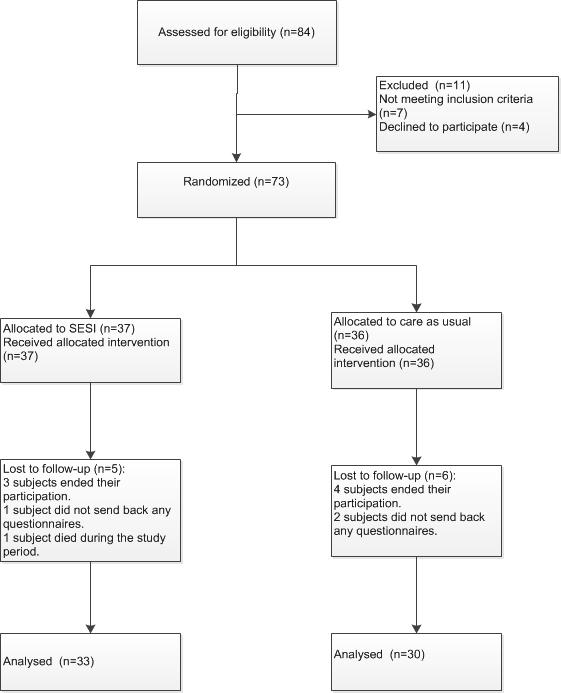

Supplement: Supplementary file 1 — Authors’ original file for figure 1 [file 12891_2014_2277_MOESM1_ESM.tiff]

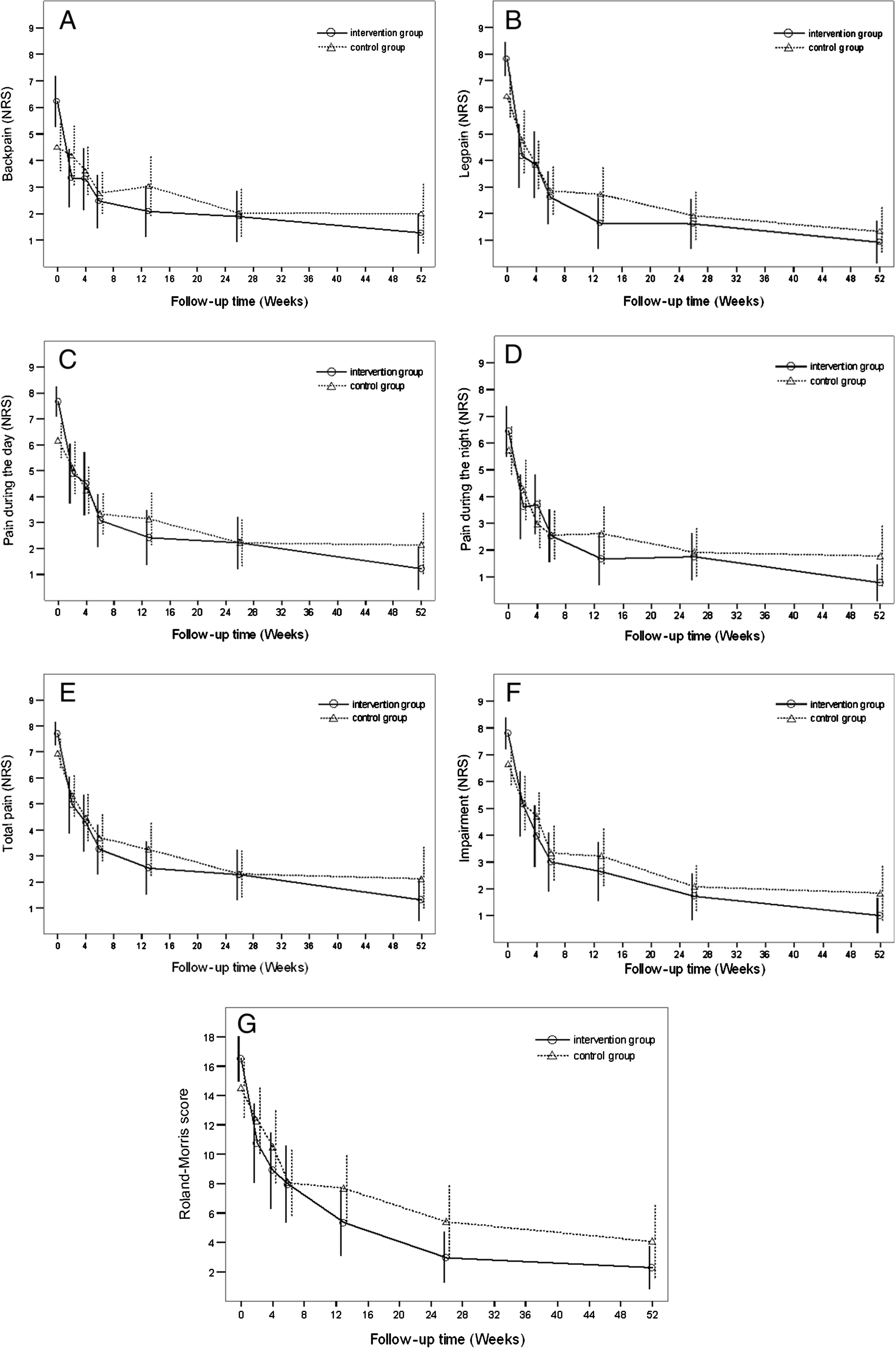

Supplement: Supplementary file 2 — Authors’ original file for figure 2 [file 12891_2014_2277_MOESM2_ESM.tif]

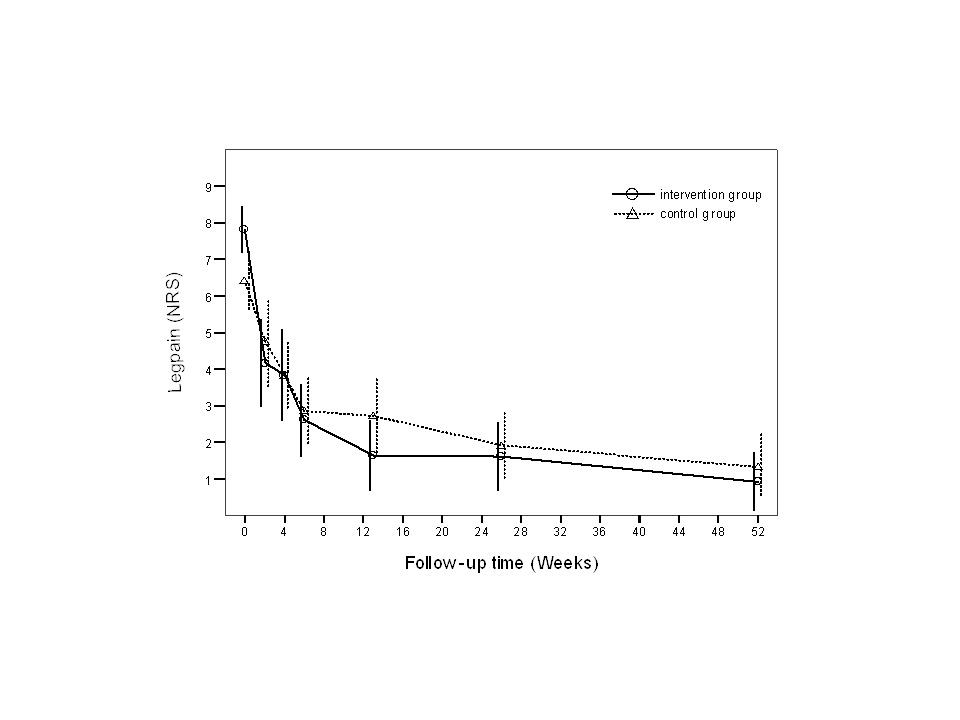

Supplement: Supplementary file 3 — Authors’ original file for figure 3 [file 12891_2014_2277_MOESM3_ESM.tiff]

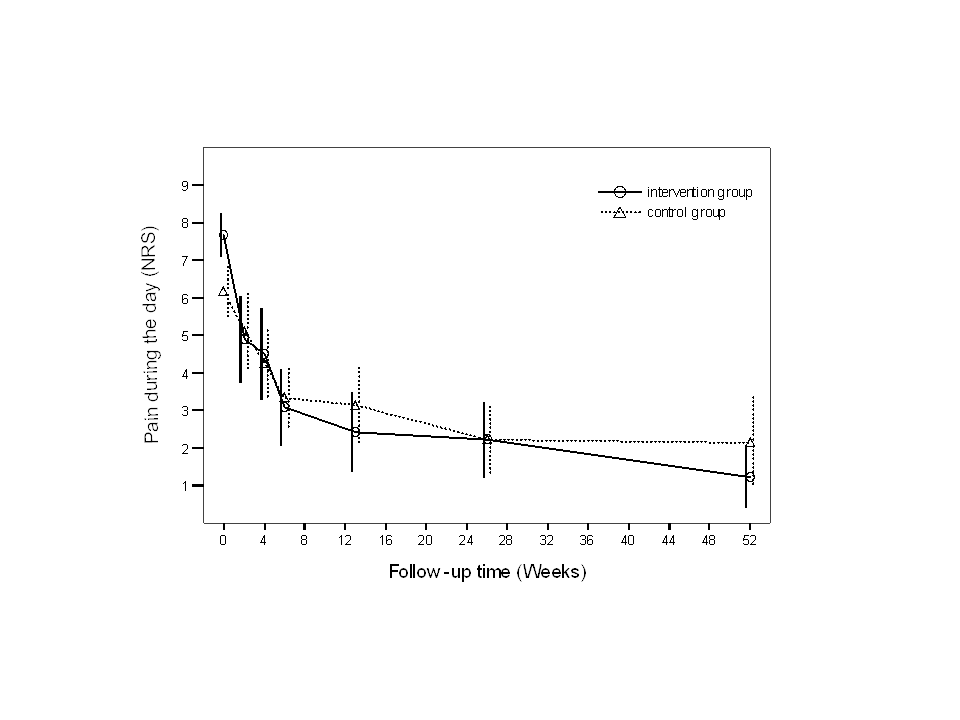

Supplement: Supplementary file 4 — Authors’ original file for figure 4 [file 12891_2014_2277_MOESM4_ESM.tiff]

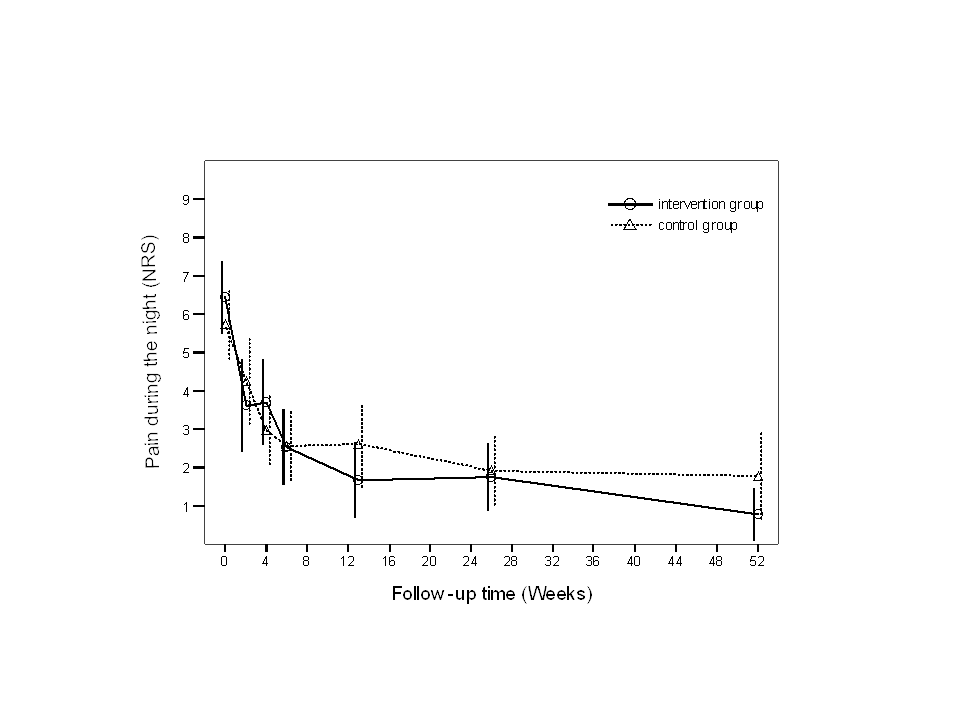

Supplement: Supplementary file 5 — Authors’ original file for figure 5 [file 12891_2014_2277_MOESM5_ESM.tiff]

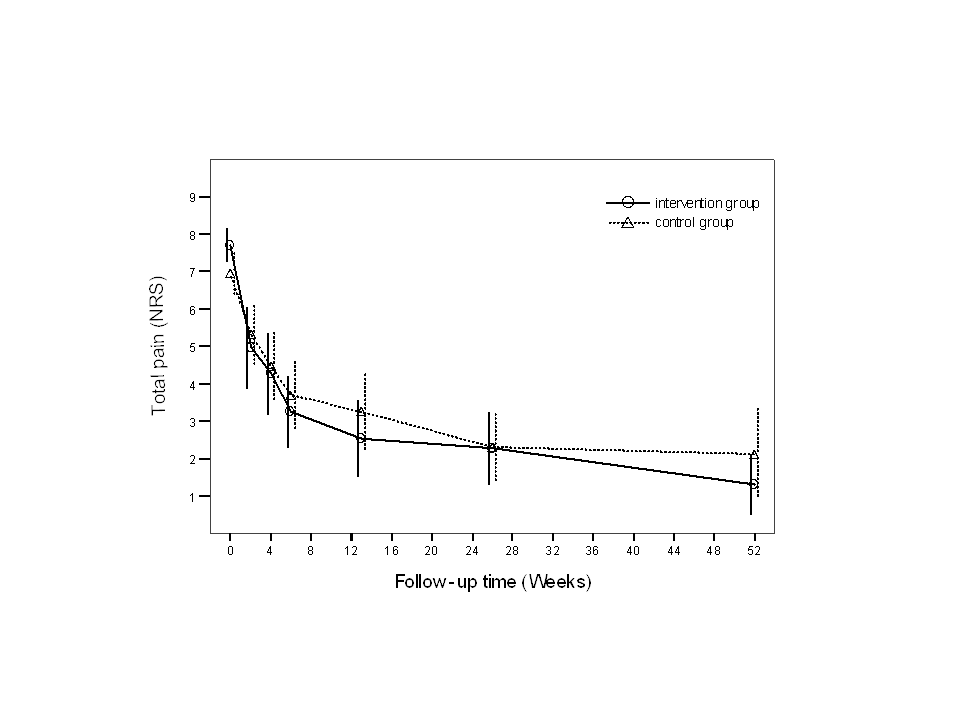

Supplement: Supplementary file 6 — Authors’ original file for figure 6 [file 12891_2014_2277_MOESM6_ESM.tiff]

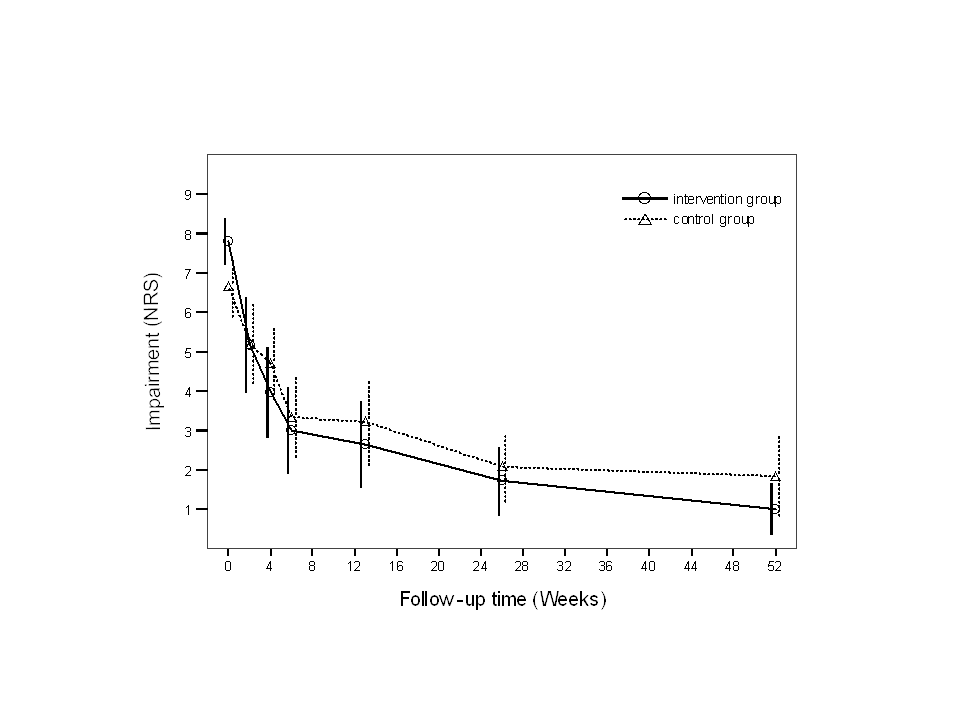

Supplement: Supplementary file 7 — Authors’ original file for figure 7 [file 12891_2014_2277_MOESM7_ESM.tiff]

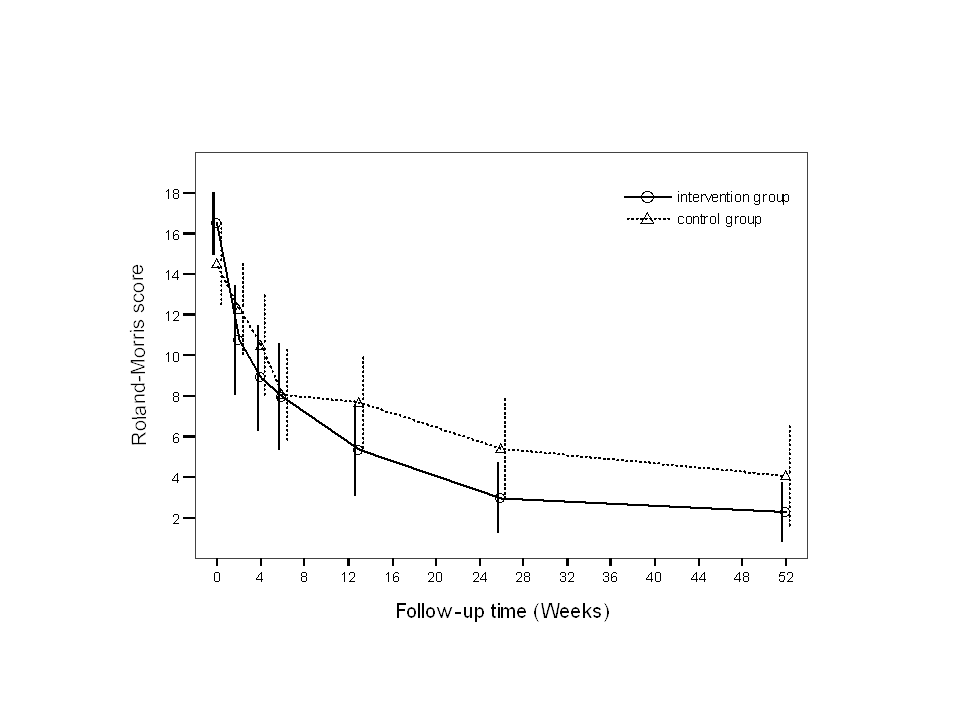

Supplement: Supplementary file 8 — Authors’ original file for figure 8 [file 12891_2014_2277_MOESM8_ESM.tiff]
